# NLRP3 promotes autophagy of urate crystals phagocytized by human osteoblasts

**DOI:** 10.1186/ar4365

**Published:** 2013-11-01

**Authors:** Isabelle Allaeys, François Marceau, Patrice E Poubelle

**Affiliations:** 1 Centre de Recherche en Rhumatologie et Immunologie (CRRI), Centre de Recherche du CHU de Québec, Department of Medicine, Université Laval, Québec, Canada; 2 CRRI, 2705, Boulevard Laurier, Québec, QC G1V 4G2, Canada

## Abstract

**Introduction:**

Monosodium urate (MSU) microcrystals present in bone tissues of chronic gout can be ingested by nonprofessional phagocytes like osteoblasts (OBs) that express NLRP3 (nucleotide-binding domain and leucine-rich repeat region containing family of receptor protein 3). MSU is known to activate NLRP3 inflammasomes in professional phagocytes. We have identified a new role for NLRP3 coupled to autophagy in MSU-stimulated human OBs.

**Methods:**

Normal human OBs cultured *in vitro* were investigated for their capacity for phagocytosis of MSU microcrystals by using confocal microscopy. Subsequent mineralization and matrix metalloproteinase activity were evaluated, whereas regulatory events of phagocytosis were deciphered by using signaling inhibitors, phosphokinase arrays, and small interfering RNAs. Statistics were carried out by using paired or unpaired *t* tests, and the one-way ANOVA, followed by multiple comparison test.

**Results:**

Most of the OBs internalized MSU in vacuoles. This process depends on signaling via PI3K, protein kinase C (PKC), and spleen tyrosine kinase (Syk), but is independent of Src kinases. Simultaneously, MSU decreases phosphorylation of the protein kinases TOR (target of rapamycin) and p70S6K. MSU activates the cleavage of microtubule-associated protein light chain 3 (LC3)-I into LC3-II, and MSU microcrystals are coated with GFP-tagged LC3. However, MSU-stimulated autophagy in OBs absolutely requires the phagocytosis process. We find that MSU upregulates NLRP3, which positively controls the formation of MSU-autophagosomes in OBs. MSU does not increase death and late apoptosis of OBs, but reduces their proliferation in parallel to decreasing their competence for mineralization and to increasing their matrix metalloproteinase activity.

**Conclusions:**

MSU microcrystals, found locally encrusted in the bone matrix of chronic gout, activate phagocytosis and NLRP3-dependent autophagy in OBs, but remain intact in permanent autophagosomes while deregulating OB functions.

## Introduction

Uric acid is an obligatory physiologic breakdown product of purine metabolism. This compound is soluble in the cytosol of cells and in plasma. However, uric acid in the extracellular milieu and tissues rapidly crystallizes because of its very low water solubility. Elevated blood uric acid is associated with several pathologies, the most representative being gout, but also hypertension, metabolic syndrome, and renal disease [1,2]. Interestingly, uric acid cannot always be considered deleterious because it has been recognized as an antioxidant, at least *in vitro,* although this effect seems uncertain *in vivo*[3-5]. Uric acid and monosodium urate (MSU) microcrystals released by injured and dying cells can be considered endogenous danger signals because they have been shown to stimulate maturation and functions of dendritic cells [6]. In addition, extracellular MSU secondary to cell injury and autoinflammatory diseases has been shown to stimulate the NLRP3 (nucleotide-binding domain and leucine-rich repeat region containing family of receptor protein 3) inflammasome [7-9]. Interestingly, although the heterocyclic chemical compound monosodium urate has no specific receptor, it can activate cells in different ways. MSU microcrystals can interact opportunistically with different receptors like CD14, CD16, and TLR-2/TLR-4, leading to intracellular signals in macrophages and neutrophils [10-12]. The same crystals were also shown nonspecifically to bind to dendritic cell-surface lipids with activation of immunoreceptor tyrosine-based motifs and subsequent recruitment of spleen tyrosine kinase (Syk) and PI3K activation [13]. Moreover, these different pathways of cell activation by MSU are followed by phagocytosis of the solid particles.

Phagocytosis, a process of endocytosis or internalization of particles, is aimed at eliminating cell debris, microorganisms, and foreign bodies in multicellular organisms. This primary major function is mainly devoted to professional phagocytes like macrophages, neutrophils, and dendritic cells. However, other cell types, like fibroblasts and osteoblasts, are competent in this respect [14-16]. Osteoblasts (OBs) can be considered nonprofessional phagocytes that are capable of internalizing different types of particles, like titanium and other small particles of biomaterials used in medical implants, latex, and various microbial pathogens [15,17-21]. They are also able to ingest MSU, leading to the production of inflammatory mediators and modifications of their functional phenotype [22]. Although specific signaling can differ, depending on the types of receptors activated by particles [23,24], the major pathways associated with phagocytosis by the professional phagocytes include the Src-family tyrosine kinases (Hck, Lyn, Fgr, Fyn, Lck), Syk, and PI3K. Interestingly, MSU interaction with neutrophils was shown to be associated with a diversified and distinct pattern of protein tyrosine phosphorylation [25-27]. MSU was also shown to activate different signaling pathways in mononuclear phagocytes like ERK-1/ERK-2, p38 MAPK, NF-κB, and AP-1 [28]. However, signaling pathways activated by MSU internalization in OBs remain unknown. It is also relevant that the bone matrix close to MSU deposits was shown to be irregularly calcified, that MSU microcrystals were abundant in new bone matrix, and that these events are associated with a low density of OBs dispersed on the osteoid [29]. As a corollary, MSU crystals in the extracellular milieu could lead to different sequences of cell activation, such as initial nonspecific contact with cell membrane, and/or opportunistic occupancy of various receptors with subsequent activation of intracellular signals that lead to their phagocytosis. It is important that phagocytosis has been linked to another highly conserved process involved in the destruction of foreign particles present in the cytosol and named autophagy [30,31].

Eukaryotic cells, to maintain their homeostasis, have lysosomes that are primary organelles with the capacity for degrading waste products and cell debris. Unfavorable conditions of life require that these cells can adapt their lysosomal responses of degradation. Autophagy (process of self-eating) is one of these adaptive responses by which cells can remove damaged or unwanted intracellular substances [32]. Thus, this housekeeping function allows the turnover of long-lived proteins, of cytoplasmic organelles, as well as of pathogens, and is related to cellular functions during nutrient starvation, cell death, repair, and infection [33]. Intracellular components to be degraded through activation of macroautophagy are first engulfed in double-membrane vesicles, named autophagosomes, before being fused with the lysosomal membrane and eventually cleared [34].

In humans, the microtubule-associated protein light chain 3 (LC3) is generated as a precursor immediately transformed into its cytosolic unconjugated form, LC3-I, which is then conjugated to the membrane phospholipid phosphatidylethanolamine to form LC3-II. This lipidated membrane-bound LC3-II is localized to preautophagosomes and autophagosomes. The amount of LC3-II correlates with the number of autophagosomes and has an apparent molecular mass smaller than that of LC3-I [35]. Thus, the evaluation of LC3-I cleavage into LC3-II reflects the activation of autophagy [36]. Although autophagy is highly regulated, the serine/threonine protein kinases TOR (target of rapamycin) appear key factors that tightly repress autophagy in yeast and mammalian cells. TOR negatively regulates the activity of Atg1, a protein kinase fundamental for autophagy, and the recruitment of LC3 [37,38]. In addition, the PI3Ks are implicated in the suppression of autophagy by acting upstream of TOR [39]. The majority of cell types have this primary function of autophagy. Deregulated autophagy has been associated with human diseases and represents a potential target for new therapeutic strategies [40].

Cell homeostasis is characterized by a low level of autophagy. Stress conditions activate the diverse and complex mechanisms of autophagy in a tightly regulated manner [41]. In addition, autophagy-generated products have been linked to innate and adaptive defenses [42,43].

Although OBs have been shown to express NLRP-3 required for caspase-1 activation associated with OB death in response to infection [44], we find that MSU activates NLRP3 in human OBs with no production of pro-IL-1β or IL-1β. We identified a new role for NLRP3 in MSU-induced autophagy in these bone cells. In OBs, MSU upregulates NLRP3, which is a positive regulator of the formation of MSU-autophagosomes. Phagocytosis of MSU by OBs is a prerequisite process to MSU-induced autophagy. However, signaling pathways of phagocytosis by OBs are not similar to those of professional phagocytes. In addition, OBs stimulated by MSU reduce their proliferation rate without change of their viability, and MSU crystals remain intact inside OBs. Together with the bone matrix irregularly calcified and the reduced number of OBs present on the osteoid close to MSU deposits [29], the present results indicate that MSU microcrystals, when phagocytized by the nonprofessional phagocyte OBs, activate NLRP3, which in turn upregulates a nonproductive macroautophagy that fails to clear MSU. Reduced anabolic functions and increased catabolic functions of OBs subsequent to MSU phagocytosis also suggest that MSU-activated OBs can be responsible for reduction of calcified bone matrix and increase of matrix degradation. Moreover, inefficient phagocytosis and autophagy of these MSU microcrystals lead to their persistent presence in autophagosomes without degradation.

## Methods

### Reagents

The incubation media α − MEM, FBS, and penicillin/streptomycin were purchased from Wisent Inc. (St-Bruno, QC, Canada). Triclinic MSU microcrystals were kindly provided by Dr R. De Médicis (University of Sherbrooke, Sherbrooke, QC, Canada) and were used under sterile pyrogen-free conditions. The mean size of the MSU microcrystals used was 10 × 1.25 μm, as determined by scanning electron microscopy. MSU was suspended at 10 mg/ml in α-MEM supplemented with 10% FBS. Accutase was from eBioscience (San Diego, CA, USA). Calcein-AM, propidium iodide (PI), cell-tracker orange CMTMR, lipofectamine and Trizol were purchased from Invitrogen Canada (Burlington, ON, Canada). Colchicine, cytochalasin D, SB203580, PD98069, 3-methyladenine, spautin-1, dynasore, and alizarin red S (ARS) were obtained from Sigma Chemical Co. (St. Louis, MO, USA). Piceatannol, wortmannin, LY4294002, Gö6979, β-glycerophosphate, and 4-amino-5-(4-chlorophenyl)-7-(*t*-butyl)pyrazolo[3,4-*d*]pyrimidine (referred to as PP2) were from Calbiochem (San Diego, CA, USA). GF109203X was from Biomol International Lp (Plymouth Meeting, PA, USA). The IκB antibodies were from Cell Signaling Technology (Danvers, MA, USA). The rabbit polyclonal anti-pro-IL-1β antibody was from Santa Cruz Biotechnology (Santa Cruz, CA, USA). IL-1β was assessed by using the DuoSet ELISA Development kit (DY201; R&D Systems, Minneapolis, MN, USA). The mouse monoclonal anti-NLRP3 (NLRP3-a, or -b) antibody and the rabbit polyclonal anti-LC3B antibody were from Novus Biologicals (Littleton, CO, USA).

#### Cell preparation

All volunteers signed a consent form that included participation to the present study and publication of the results in accordance with the Declaration of Helsinki. The institutional review board of the Université Laval approved the study. Primary OB cell cultures were prepared from human trabecular bone explants obtained from female or male subjects (50 to 65 years old) undergoing orthopedic surgery for degenerative joint diseases. None of the volunteers had metabolic bone disorders or malignancy. Explants and subsequent conditions of culture were as previously described [22,29,45,46]. In brief, OBs were grown in α-MEM supplemented with 10% FBS. The medium was replaced every 3 days until cellular confluence. At confluence, bone explants were transferred to new six-well plates to allow remaining OBs to migrate and adhere to the plate. Human OBs were recovered by using the enzyme Accutase and plated at starting densities of 0.5 to 1 × 10^6^ cells/well in α-MEM with 10% FBS. All incubations were performed at the first cellular passage and at 80% to 90% cellular confluence. OBs were all incubated in medium with antibiotics (1% pen-strep) at 37°C in a humidified atmosphere containing 5% CO_2_.

#### Evaluation of phagocytosis

Confluent OBs were stimulated 24 hours, 48 hours, or 3 or 7 days with MSU at 0.5 mg/10^6^ cells and analyzed with optic microscopy. To quantify phagocytic vacuoles at 24 hours, five pictures randomly located in the well were analyzed, and vacuoles containing MSU were numbered with a cell counter and Image J software (NIH). Pharmacologic studies of MSU phagocytosis by OBs used optimal concentrations of colchicine, cytochalasin D, SB203580, PD98069, piceatannol, wortmannin, LY4294002, Gö6979, GF109203X, and PP2, according to previous publications [47-55].

#### Viability

Confluent OBs were stimulated with 0.3, 0.5, or 1 mg MSU/10^6^ cells for 24, 48, or 72 hours. Cells were washed with PBS and then detached by using Accutase (500 μl/well) 10 minutes at 37°C. Necrotic and late apoptotic cells were identified by PI incorporation (5 μg/ml) and evaluated with cytofluorometry. Cells that did not incorporate PI have intact membranes and were considered viable cells [56].

#### Proliferation assay

OB proliferation was evaluated by using the CellTiter 96 A_queous_ One Solution Cell Proliferation Assay, as specified by the Promega manufacturer’s protocol (Madison, WI, USA). In brief, 1,500 cells were plated in 96-well plates on day 1 for 24 hours in 100 μl of α-MEM containing 10% FBS, and then starved on day 2 with 100 μl of α-MEM containing 0.1% FBS for 24 hours. On day 3, cells were stimulated for 96 hours with vehicle or with different concentrations of MSU in 100 μl of α-MEM containing 10% FBS. After 96 hours, 20 μl of CellTiter 96 A_queous_ One Solution Reagent (containing a tetrazolium compound) were directly added to the culture wells. Cells in the presence of the reagent were further incubated for 3 hours at 37°C in a 5% CO_2_ humidified atmosphere, and then the absorbance was recorded at 490 nm. The quantity of formazan product (from the bioreduction of MTS tetrazolium compound by cells) corresponding to the optical density at 490-nm absorbance is directly proportional to the number of living cells in culture.

#### Confocal microscopy

Confluent OBs were stained with 2 μ*M* CMTMR (30 minutes, 37°C) and then stimulated with 0.5 mg of MSU for 48 hours at 37°C. Confocal microscopy analyses were performed with Olympus Fluoview 300 microscope by using differential interference contrast (DIC) and helium-neon (543 nm) lasers, magnification × 400.

#### Evaluation of mineralization

Mineralization of cell cultures was evaluated by alizarin red S (ARS) staining. OBs were seeded at 2 × 10^5^ cells/well in six-well tissue-culture dishes and maintained in α-MEM, 10% FBS supplemented with 10 m*M* β-glycerophosphate, at 37°C in a humidified atmosphere containing 5% CO_2_. Culture medium was replaced every 3 days until day 20. OBs were treated with MSU or vehicle at day 8 (90% confluence). At day 20, cells were fixed for 20 minutes with buffered formalin and then stained for 20 minutes with 40 m*M* ARS, pH 4.0 to 4.2 at room temperature. After four washes with distilled H_2_O, ARS was extracted, as previously described [57]. In brief, ARS cells were incubated 30 minutes with acetic acid and then heated 10 minutes at 85°C; pH was restored at 4.2 with NaOH, and ARS absorbance was read at 405 nm.

#### MMP activity

Evaluation of generic matrix metalloproteinases (MMP) was assessed with the SensoLyte Generic MMP assay kit (Anaspec, San Jose, CA, USA) that detects the activity of a variety of MMPs, including MMP-1, 2, 3, 7, 8, 9, 12, 13, and 14. Five-FAM (fluorophore) and QXL520(quencher), labeled FRET peptide substrates, were used for continuous measurement of the enzyme activities. On the cleavage of the FRET peptide by MMPs, the fluorescence of 5-FAM was recovered and monitored at excitation/emission wavelengths of 490 nm/520 nm. Confluent OBs were treated 24 hours with or without 0.5 mg MSU in α-MEM containing 1% FBS. Medium was then centrifuged 2 minutes at 10,000 rpm, and 50 μl of supernatant was added to 50 μl of MMP substrate for 20 minutes. MMP activity in MSU-stimulated cells was compared with MMP activity in untreated cells.

#### RNA isolation and real-time PCR

OB total RNA was isolated by using Trizol (Invitrogen Lifetechnology). In brief, around 10^6^ confluent cells, stimulated with MSU or vehicle, were washed in PBS and then homogenized in 1 ml Trizol. Total RNA was then extracted, according to the manufacturer’s protocol. Reverse-transcription and real-time PCR were performed essentially as previously described in [58]. In brief, first-strand cDNA synthesis was performed by using 1 μg of total RNA with Superscript II (Invitrogen Lifetechnology) in recommended conditions, with 10 ng of random hexamers. Amplification of osteoblast cDNA was carried out in a Rotor-Gene 3000 operated with Rotor Gene software version 6.0.19 (Corbett Reasearch, Mortlake, 2137 NWS, Australia). Each sample consisted of: 50 ng cDNA, 1.3 m*M* MgCl_2_, 0.2 m*M* dNTP, 500 n*M* primers, 0.5 unit of Taq polymerase (GE Healthcare Bio-Sciences AB, Uppsala, Sweden), and Sybr Green dye (Molecular Probes, Eugene, OR, USA; 1/30,000 dilution) in a reaction volume of 20 μl.

Amplification conditions were as follows: 95°C (20 seconds), 60°C (20 seconds), 72°C (20 seconds); 35 cycles. Specificity of each reaction was ascertained by performing the Melt procedure (58°C to 99°C; 1°C/5 seconds) after completion of the amplification protocol, according to the manufacturer’s instructions. Primers used in real-time PCR procedures were designed with Primer 3 software as GAPDH: 5′-CGAGATCCCTCC-AAAATCAA-3′ (forward), 3′-TTCACACCCATGACGAAC AT-5′ (reverse); procollagen-α1: 5′-ACGTCCTGGTGAAGTTGG-TC-3′ (forward), 3′-CAGGGAAGCCTC-TCTCTCCT-5′ (reverse).

#### Proteome profiler assay

Signaling pathways were investigated by using the Proteome Profiler arrays (R&D Systems, Minneapolis, MN, USA). The Human Phospho-Kinase array is a nitrocellulose membrane where antibodies against 46 kinase phosphorylation sites have been spotted in duplicate. Cell lysates from untreated, 5 minute, 20 minute, and 1 hour MSU-activated cells were prepared in lysis buffer provided with the proteome profiler (R&D). In total, 250 μg of protein was used for each array and incubated with the nitrocellulose membrane array overnight at 4°C. The array was washed and then incubated with a cocktail of phospho-site-specific biotinylated antibodies for 2 hours at room temperature, and washed before adding Streptavidin–HRP for 30 minutes. Signals were developed with an enhanced chemiluminescence Western blotting detection system and recorded on x-ray film. Densities of individual dots corresponding to a phosphorylated kinase were measured by Image J software, and a comparison between untreated and MSU-activated samples was performed.

#### Immunoblot analysis

After incubation, around 5.10^5^ confluent adhering OBs were washed with PBS and then directly lysed in Laemmli buffer. Cells were boiled for 10 minutes. Samples were subjected to 15% SDS-polyacrylamide gel electrophoresis (SDS-PAGE) and transferred to Immobilon membranes (Millipore Corporation, Billerica, MA, USA). Equal protein loading and transfer efficiency were visualized with β-actin evaluation. Membranes were saturated for 30 minutes at room temperature in Tris-buffered saline (TBS, 25 m*M* Tris–HCl, pH 7.6, 0.2 *M* NaCl) with 0.5% Tween 20, containing 5% (wt/vol) dried milk, and subsequently exposed overnight at 4°C to the LC3-B rabbit polyclonal antibody, NLRP-3b mouse monoclonal antibody, P-IκB or IκB mouse antibodies, or 1 hour at room temperature to the actin mouse monoclonal antibody. Membranes were washed twice in TBS-Tween and incubated with secondary antibodies. Bounded antibodies were revealed with the enhanced chemiluminescence Western blotting detection system after TBS-Tween washes, as specified by the manufacturer’s protocol (Pierce Biotechnology, Thermo Fisher Scientific, Rockford, IL, USA).

#### LC3-GFP transfection

OBs (90% confluence) were transfected with LC3-GFP plasmid (gift from Dr T. Yoshimori, Osaka University, Japan) for 24 hours by using lipofectamine, according to the manufacturer’s protocol. After 4 hours of MSU stimulation, cells were then observed with confocal microscopy (Olympus Fluoview 300 microscope using a helium-neon (488 nm) laser, ×400 magnification).

#### Small interfering RNA knockdown of NLRP3 expression

Knockdown of NLRP3 expression was achieved by transfecting OBs with a combination of two small interfering RNAs (siRNAs) against NLRP3 or AllStars Negative Control siRNA (Qiagen Inc., Mississauga, ON, Canada). Predesigned siRNAs against NLRP3 target sequences were SI02634009 and SI02634030. OBs were transfected with these siRNAs in the presence of HiPerFect Transfection Reagent by following the manufacturer’s protocol (Qiagen). After 24 hours of transfection, knockdown of NLRP3 protein expression was confirmed with immunoblot, and these cells were stimulated or not with 0.5 mg MSU for 8 hours.

#### Densitometric analyses

Immunoblots were analyzed by using ImageJ software (Image Processing and Analysis in Java, NIH) to quantify band intensity assessed with densitometry. Results are presented as mean values of arbitrary densitometric units normalized to the expression of β-actin or as levels in MSU-stimulated cells over levels in unstimulated cells.

#### Statistics

Results are expressed as mean ± SEM. Statistical analyses were performed by using GraphPad Instat 3.0 (GraphPad Software Inc., San Diego, CA, USA). Two groups were analyzed by using paired or unpaired *t* tests. For three groups and more, statistical analyses were performed by using the one-way ANOVA Bonferroni multiple-comparison test or the repeated measures ANOVA, followed by Tukey multiple-comparison test. Significance was set at *P* < 0.05.

## Results

### Human osteoblasts internalize MSU

OBs are known to ingest MSU microcrystals *in vitro* with some efficacy [22]. These observations, together with the pathologic findings of MSU included in bone matrix and a scarce presence of OB close to tophaceous bone lesions [29], suggest that OBs are unable to destroy these crystals. Thus, MSU could remain intact inside OBs and deregulate specialized functions of OBs.

**Figure 1:**
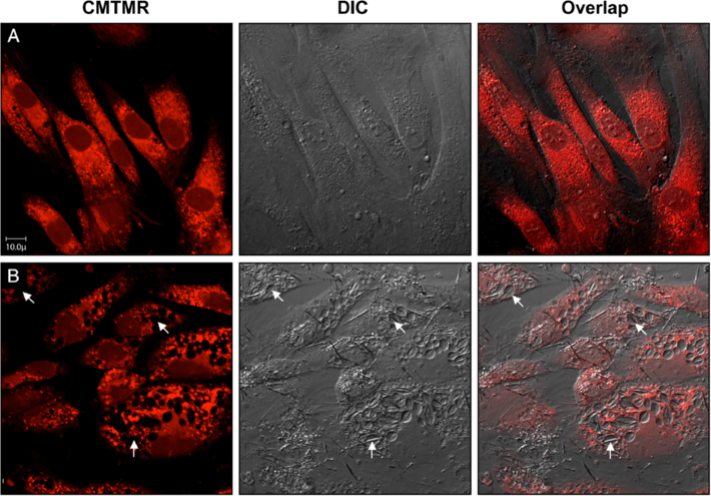
**Phagocytosis of monosodium urate by osteoblasts.** Confluent human OBs, previously stained with CellTracker Orange CMTMR, were cultured with vehicle **(A)** or with 0.5 mg MSU/10^6^ cells **(B)** for 48 hours at 37°C, and then analyzed with confocal microscopy by using Olympus Fluoview microscope with helium-neon 543-nm lasers (left panel), differential interference contrast (DIC; middle panel), and overlap (right panel); ×400 magnification. Arrows indicate vacuoles and crystals of MSU. Representative of independent experiments with OB from three different donors.

To evaluate the fate of MSU in the presence of OBs, live confluent primary human OBs were cultured with graded concentrations of MSU during 7 days. OBs that phagocytized MSU showed, after 48 hours of incubation, consistent morphologic changes, as studied with confocal microscopy. OBs dose-dependently internalized MSU from 0.1 to 1 mg/10^6^ cells with an optimal effect at 0.5 mg/10^6^ cells, followed by a plateau (data not shown). More than 90% of OBs had MSU internalized in large and fluid-filled vacuoles, each containing a single microcrystal (Figure 1). Volume and shape of vacuoles depend on crystal size. Vacuoles were individualized with light microscopy after, at least, 24 hours of incubation. Numbers of vacuoles with MSU averaged 30 per OB. Most of MSU were completely internalized in cells, but some crystals remained partially engulfed or alongside the membrane. After 7 days of culture, phagocytosis of 0.5 mg MSU/10^6^ OBs was associated with unchanged vacuoles (data not shown). These data suggest a prolonged process that could partly detoxify the cells by retaining MSU microcrystals in permanent phagosomes with a final noncapacity of OB to eliminate MSU-containing vacuoles.

### MSU affects OB proliferation but not viability

**Figure 2:**
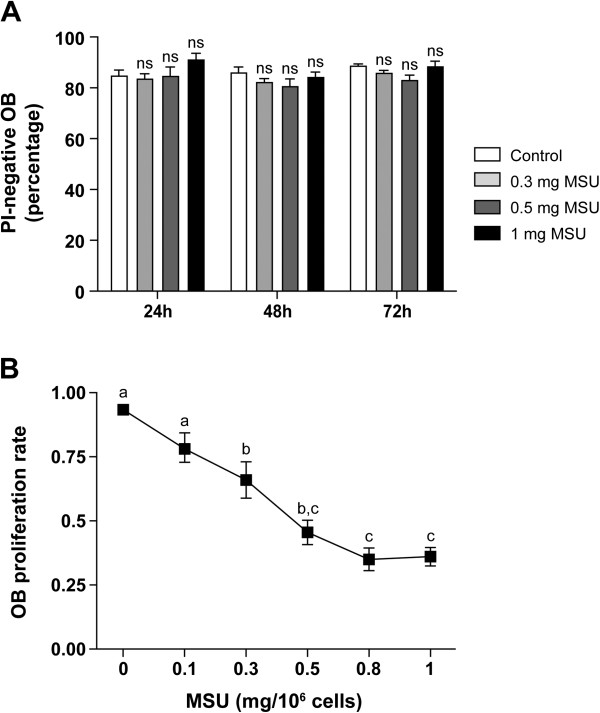
**Effects of MSU on OB viability and proliferation. (A)** Confluent OBs were incubated with vehicle (control) or with MSU from 0.3 to 1 mg/10^6^ cells for 24, 48, or 72 hours. Cells were washed with PBS and removed with Accutase. Propidium iodide (PI) exclusion was analyzed with cytofluorometry. **(B)** Cells were plated 3 days with vehicle or with MSU in 96 wells and then analyzed with the CellTiter 96 AQ_ueous_ One proliferation assay. Data are represented as a ratio of MSU-stimulated to vehicle-stimulated cells. Results are given as mean ± SEM of values from four **(A)** or three **(B)** different donors. Statistical analysis: one-way ANOVA followed by Bonferroni multiple-comparison test: ns, nonsignificant; means without a common letter differ: *P* < 0.05.

Because MSU can modulate cellular apoptosis and proliferation [59,60], the impact of MSU on OB survival and proliferation was evaluated before studying specialized OB functions. MSU at concentrations up to 1 mg/10^6^ cells for 72 hours of culture did not modify the incorporation of propidium iodide (PI) by OBs, and an average of 80% PI-negative OBs was routinely obtained in control conditions, as well as in the presence of MSU (Figure 2A). In contrast, the proliferation rate of MSU-treated OBs dose-dependently decreased from 0.1 to 1 mg MSU/10^6^ cells (Figure 2B). The significant threshold reduction was observed at 0.3 mg MSU, with a plateau of reduction attained at 0.8 mg MSU. The respective proliferation rates were reduced from 30% to 55% of the OB proliferation rate in control conditions. Thus, although MSU microcrystals at the concentrations tested did not modify the viability of OBs, they significantly decreased the proliferation of OBs and could, in parallel, affect other functions.

### MSU alters OB functions

#### Mineralization

**Figure 3:**
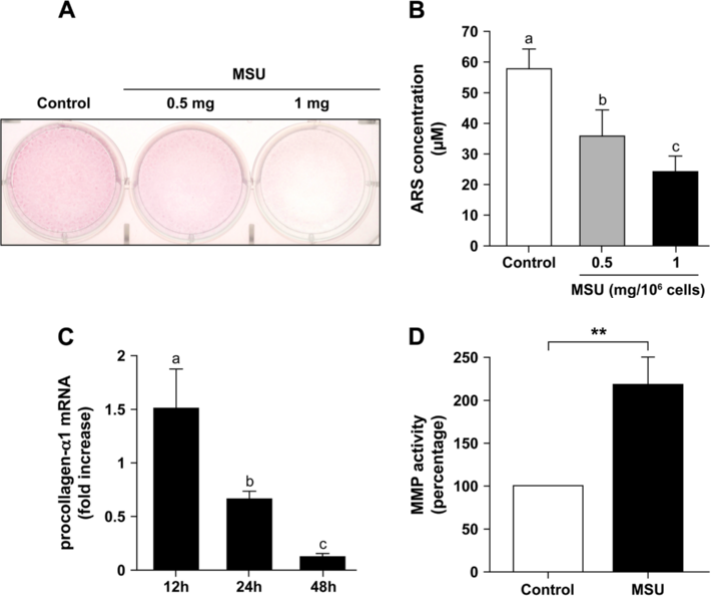
**OB functions of matrix mineralization, procollagen-α1 expression, and matrix metalloproteinase (MMP) activity. (A, B)** Mineralization *in vitro* by normal human OBs. Cells were cultured 20 days in the presence of 10 m*M* β-glycerophosphate and stimulated with vehicle (control) or with 0.5 and 1 mg MSU for 12 days before alizarin red staining (ARS). **(A)** Representative ARS staining in vehicle- or MSU-stimulated OBs. **(B)** Quantification of ARS, values are shown as mean ± SEM from three different donors. **(C)** Expression of procollagen-α1 by OBs stimulated with MSU. Cells were incubated with vehicle or with 1 mg MSU for 12, 24, and 48 hours. Gene expression was evaluated with QRT-PCR. Results were GAPDH-normalized and expressed as procollagen-α1 expression by MSU-stimulated OBs relative to unstimulated cells (fold increase). **(D)** Evaluation of generic MMP activity. Confluent OBs were incubated with vehicle (Control) or with 0.5 mg MSU for 24 hours, and MMP activity in the supernatant was evaluated according to the manufacturer’s protocol. MMP activity in MSU-stimulated cells was calculated as the ratio of MSU-stimulated cells to vehicle-treated cells (control referred to as 100%). Statistics: **(B)** and **(C)** were analyzed by using one-way ANOVA followed by Tukey multiple-comparison test (*n* = 4 different donors); means without a common letter differ: *P* < 0.05. **(D)** Absorbance values were compared by using paired two-tailed *t* tests. **P = 0.002 (*n* = 5 different donors).

MSU present in the culture medium of human OBs affects parameters implicated in bone mineralization, such as alkaline phosphatase activity and osteocalcin content [22]. To assess the mineralization function of OBs in the presence of MSU or vehicle *in vitro*, OB cultures were stained with alizarin red S (ARS), a marker of matrix calcium that allows a quantitative evaluation of mineralization [57,61]. OBs incubated with MSU showed a reduced ARS staining of the newly calcified matrix (Figure 3A). The quantities of ARS in cultures of MSU-activated OBs were dose-dependently decreased by 1.6- and 2.1-fold compared with those observed in vehicle-treated OBs (Figure 3B). Moreover, the addition of MSU suppressed in a time-dependent manner the expression of the mRNA of procollagen-α1, a typical bone-matrix constituent, with a sixfold decrease at 48 hours in the presence of 1 mg MSU (Figure 3C). These data indicate that MSU affects the formation of certain matrix components and in fine bone matrix mineralization.

#### MMP activity

Bone matrix degradation depends, among other factors, on enzymes such as matrix metalloproteinases (MMPs) that are known to be implicated in pathophysiological processes [62]. Although bone-matrix degradation is related mainly to osteoclasts, OBs can also be involved in bone resorption through their production of several MMPs [63-65]. The activity of generic MMPs, as evaluated in supernatants of OBs cultured with MSU, was increased by 120% over that of unstimulated cells (Figure 3D). These results indicate that MSU-stimulated OBs may be directly implicated in matrix degradation of bone with MSU deposits.

### Phagocytosis of MSU by OBs is tightly regulated

#### Signaling pathways affected by MSU

**Figure 4:**
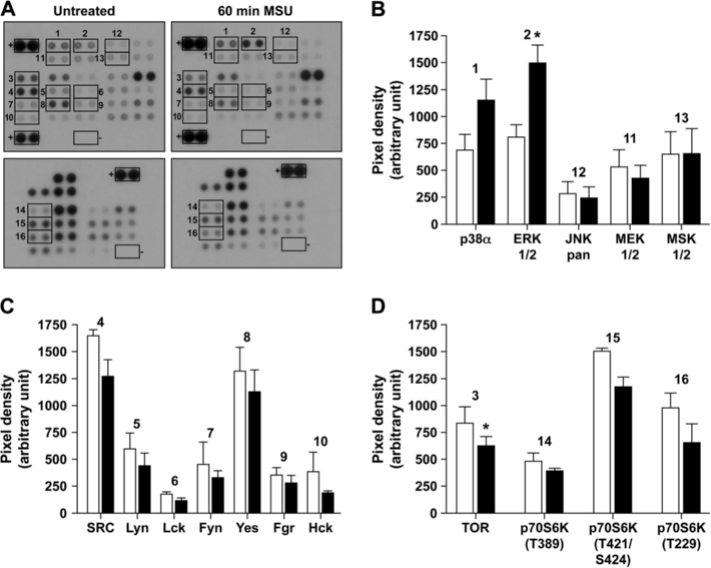
**Phosphoproteomic analysis of MSU-stimulated OBs.** The Human Phospho-Kinase Array was used to detect multiple phosphorylated kinases in OBs, either untreated or stimulated with 1 mg MSU for 60 minutes. **(A)** Template showing the location of kinase antibodies spotted onto the Human Phospho-Kinase Array kit. Positive and negative controls are indicated by **+** and **–** adjacent to appropriate spots. Signals of relevant kinases in response to MSU stimulation are indicated by numbers. **(B, C, D)** Quantification of mean spot pixel densities of untreated cells (white) versus MSU-stimulated cells (black). **(B)** Representation of phosphorylated MAP kinases. **(C)** Phosphorylated Src kinase family. **(D)** Phosphorylated TOR and p70S6K. Representative of independent experiments with OB from three different donors. Results are shown as mean ± SEM of densitometric values (*n* = 3). Statistical analysis was performed by using the paired two-tailed *t* test to compare phosphorylated proteins from OBs under MSU versus vehicle; *P < 0.05.

These data document profound effects of MSU on the behavior of OBs. These data indicate that the pathways regulating OB functions are likely to be affected by the presence of MSU. By using a protein kinase array that detects specific phosphorylation of 46 kinase phosphorylation sites, certain effector signaling proteins were investigated in MSU-stimulated OBs (Figure 4A). Phosphorylation levels after 1 hour of MSU stimulation were higher than those recorded at 5 and 20 minutes (data not shown). Thus, a 1-hour MSU stimulation of OBs was associated with a phosphorylation increase of p38α (T180/Y182) by 86% and ERK 1/2 (T202/Y204, T185/Y187) by 94% (Figure 4B), whereas the phosphorylation of Src kinases tended to be inhibited (Src (Y419), Yes (Y426), Hck (Y411), Fyn(Y420)) or unchanged (Fgr (Y412), Lck (Y394)) (Figure 4C). Additionally, phosphorylation of the serine/threonine protein kinases TOR and p70S6K was decreased by the presence of MSU (Figure 4D).

#### Pharmacologic modulation of phagocytosis

Considering these results on signaling pathways suggesting that MSU modulated the phosphorylation status of various kinases, the investigation was pursued to determine the role in OBs of those kinases that are known to be implicated in phagocytosis, a dynamic mechanism of endocytizing particles. The engulfment of large particles is governed by the microfilament and microtubule cytoskeletons. Therefore, the effects of cytochalasin D, an inhibitor of actin polymerization, and colchicine, an inhibitor of microtubule polymerization, were examined on MSU internalization by OBs (Table 1). Cytochalasin D pretreatment abrogated the formation of vacuoles associated with MSU phagocytosis. In contrast, colchicine did not inhibit the appearance of vacuoles containing MSU. Mechanisms underlying phagocytosis also implicate several intracellular signaling pathways that lead to cytoskeleton reorganization and ingestion of particles. From that point of view, pharmacological inhibitors can help decipher signaling pathways associated with MSU phagocytosis by OBs (Table 1). The phosphoinositide 3-kinases that control cytoskeleton dynamics, signal transduction, and membrane trafficking [66,67] were targeted by two pan-PI3K inhibitors, wortmannin and LY294002. Both inhibitors reduced by 50% the vacuole-formation process, suggesting a role of PI3K in the internalization of MSU by OBs. Protein kinase C (PKC) can also be involved in the transduction of phagocytic signals [68]. The inhibitor of pan-PKC isoforms GF109203X (GFX) and the inhibitor of classic-type PKC isoforms Gö6976 were found to reduce by approximately 60% and 70% MSU-vacuole formation, respectively, thereby supporting an involvement of PKC in this process. The extracellular kinase (ERK1/2) inhibitor PD98069 reduced by 44% the MSU-induced formation of vacuoles, confirming an implication of these MAPK in the process of vacuole formation by OBs. As Syk tyrosine kinases have been shown to control phagocytosis [25,69], the Syk inhibitor piceatannol was tested on MSU-activated OBs. Piceatannol reduced the MSU-induced formation of vacuoles by 58%, indicating an involvement of Syk kinases in this process. Surprisingly, the inhibition of Src kinases by PP2 failed to modulate the MSU-induced formation of vacuoles, whereas PP2 completely inhibited Src kinases in MSU-activated neutrophils (data not shown). Conversely, OB preincubated with the p38 MAPK inhibitor SB203580 exhibited a twofold increase of MSU-induced vacuole formation. Together, these results indicate that phagocytosis and vacuole formation by OBs in the presence of MSU are dependent, at least in part, on different types of kinases like PI3K, PKC, ERK1/2, and p38 MAPK, and Syk and are independent of Src kinases. Moreover, ERK1/2 and p38 MAPK show antagonistic effects on this process in OBs.

**Table 1:** Effect of pharmacologic inhibitors on MSU phagocytosis by OBs

	Vacuole number (statistics)*
Control	13.1 ± 3.7 (a)
+ MSU	361 ± 27 (b)
Cytochalasin D + MSU	23.2 ± 10.7 (a)
Colchicine + MSU	321 ± 60 (b)
Control	14.5 ± 3.0 (a)
+ MSU	323 ± 32 (b)
Wortmannin + MSU	157 ± 31 (c)
LY4294002 + MSU	146 ± 27 (c)
Control	16.1 ± 3.2 (a)
+ MSU	281 ± 52 (b)
GF109203X + MSU	126 ± 10 (a)
Gö6976 + MSU	95 ± 10 (a)
SB203580 + MSU	612 ± 101 (c)
PD98069 + MSU	157 ± 39 (a)
Control	16.3 ± 3.2 (a)
+ MSU	193 ± 23 (b)
Piceatannol + MSU	82 ± 20 (a)
PP2 + MSU	231 ± 42 (b)

Confluent OBs were preincubated 15 minutes with 1 μ*M* cytochalasin D or 10 μ*M* colchicine at 37°C and then stimulated with 0.5 mg MSU/10^6^ cells for 24 hours. Confluent OBs were preincubated with PI3K inhibitors: wortmannin 50 n*M* or LY4294002 10 μ*M*, with PKC inhibitors: GF109203X 5 μ*M* or Gö6976 1 μ*M*, with p38 or ERK1/2 inhibitors: SB203580 10 μ*M* or PD98069 20 μM, or with a Syk inhibitor: piceatannol 20 μ*M* and a Src inhibitor: PP2 10 μ*M*, 15 minutes at 37°C and then stimulated with 0.5 mg MSU/10^6^ cells for 24 hours. Cells were analyzed with microscopy (×200 magnification), and results expressed as cumulative data means of values ± SEM of five pictures for each donor. Statistical analysis was performed on vacuole counts by using the repeated measures ANOVA followed by Tukey multiple-comparison test (*n* = 4 different donors). *Means without a common letter differ: *P* < 0.05.

#### MSU activates autophagy in OBs

**Figure 5:**
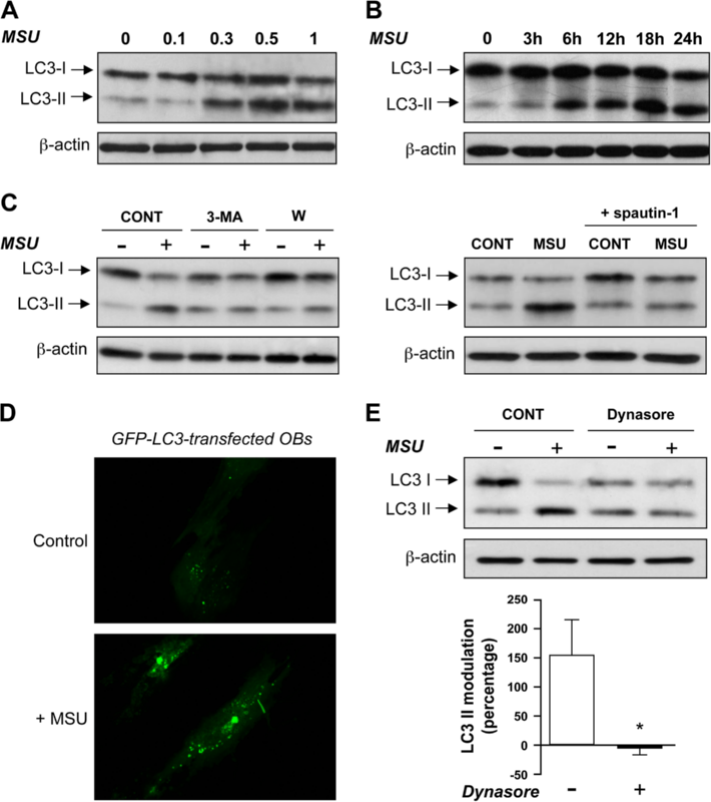
**MSU induces endogenous LC3 conversion. (A)** Expression levels of LC3-I and LC3-II after MSU stimulation. Confluent OBs were cultured in α-MEM with 10% FBS and were stimulated with 0.1 to 1 mg MSU for 24 hours. Reactions were stopped, and cells were prepared to perform immunoblot analysis with anti-LC3-I/LC3-II and anti-β-actin antibodies. **(B)** Kinetic effects of MSU. OBs were cultured as in **(A)** and stimulated by 0.5 mg MSU for the indicated times. **(C)** Effects of 3-methyladenine (3-MA), wortmannin (W), and spautin-1 on MSU-induced conversion of LC3. Cells were pretreated with vehicle (CONT), 3-MA 6 μ*M*, W 50 n*M*, or spautin-1 (10 μ*M*) for 30 minutes. The cells were then stimulated with 0.5 mg MSU for 24 hours in the absence or in the continuous presence of 3-MA, W, or spautin-1. Immunoblots are representative of four different donors (3-MA, W), and of three different donors (spautin-1). **(D)** Autophagosome localization in GFP-LC3-transfected OBs. OBs were transfected with GFP-LC3 plasmid and then incubated with vehicle (Control) or 0.5 mg MSU for 4 hours before analysis with confocal microscopy (×200 magnification). **(E)** Effects of Dynasore on MSU-induced conversion of LC3. Confluent OBs were pretreated with vehicle (CONT) or 80 μ*M* Dynasore for 10 minutes, and then incubated with vehicle or 0.5 mg MSU for 24 hours. Data are expressed as arbitrary densitometric units normalized by β-actin levels; LC3-II conversion induced by MSU was the ratio of LC3-II levels of MSU-stimulated cells over LC3-II levels of vehicle-stimulated cells. The effect of Dynasore is presented as mean value ± SEM of percentage inhibition of LC3-II conversion. Statistical analysis was performed by using the paired two-tailed *t* test (*n* = 3 different donors); *P < 0.05.

Proteome-profiler analyses revealed that the phosphorylation of TOR (S2448), as well as of the marker of TOR activity p70S6K (T389, T421/424, T229), was decreased after MSU stimulation (Figure 4D). TOR is a repressor of autophagy, and diminution in TOR phosphorylation allows autophagy [70]. Because uric acid has been found to be a danger signal [6], we hypothesized that MSU could alert OBs through an autophagic response based on these data showing that the TOR pathway was downregulated and that MSU-activated OBs reduced their proliferation without alteration of their viability. Microtubule-associated protein LC3 is an effector of macroautophagy, and its cleavage and lipidation have been used as a specific marker to monitor autophagy [71]. MSU dose- and time-dependently induced the cleavage of LC3-I into LC3-II (Figure 5A, B). In addition, preincubation of OBs with 3-methyladenine, an inhibitor of autophagic sequestration through class III PI3K [39], or with wortmannin, an inhibitor of PI3K involved in autophagy and phagocytosis [72,73], abolished the cleavage of LC3-I into LC3-II (Figure 5C). Experiments were also performed with OBs preincubated with spautin-1, an inhibitor of autophagy that targets the beclin1 subunit of Vps34 complexes [74]. Spautin-1 efficiently inhibited the cleavage of LC3-I into LC3-II in MSU-activated OBs (*P* < 0.05; *n* = 3). Moreover, the addition of MSU to OBs transfected with green fluorescent protein (GFP)-tagged LC3 showed a rapid increase of labeled vacuoles in their cytosol, as well as MSU coated with GFP-tagged LC3 (Figure 5D). These results indicate that MSU in human OBs induced endogenous LC3 conversion and stimulated the process of autophagy while they were progressively engulfed in OBs.

After our pharmacologic study (see earlier) that indicated activation of signaling pathways involved in both autophagy and phagocytosis, and because giant vacuoles containing MSU appeared comparatively late versus the rapid generation of autophagosomes, was the *primum movens* to destroy these solid particles autophagy or phagocytosis? Dynasore, a dynamin inhibitor, was used to abrogate the phagocytic pathways by blocking vesicle formation [75,76]. Interestingly, pretreatment of OBs with dynasore totally abolished the MSU-induced cleavage of LC3-I into LC3-II (Figure 5E), suggesting that phagocytosis precedes autophagy and that MSU-activated autophagy directly depends on crystal phagocytosis by OBs.

### MSU stimulates NLRP3 in OBs

**Figure 6:**
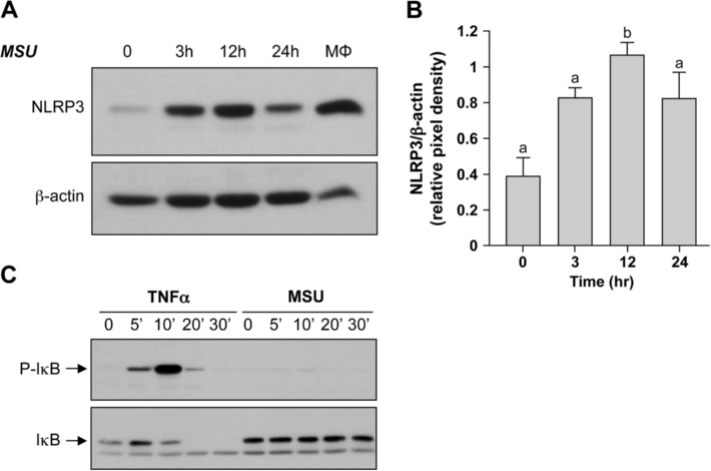
**MSU activates NLRP-3 without any effect on the NF-κB pathway. (A)** Expression levels of NLRP-3 during MSU stimulation. Confluent cells were stimulated with 0.5 mg MSU for the indicated times and then subjected to immunoblot analysis by using anti-NLRP-3 and anti-β-actin antibodies. Macrophages (MΦ) stimulated for 24 hours with LPS, 100 ng/ml, were used as positive control. **(B)** Quantification of NLRP-3 levels in MSU-stimulated OBs. Pixel-density results were normalized with β-actin, and cumulative data are expressed as mean ± SEM (three different donors). Statistical analysis was performed by using the one-way ANOVA Bonferroni multiple-comparison test; means without a common letter differ: *P* < 0.05. **(C)** Analysis of the NF-κB pathway activation. Confluent OBs were stimulated with 0.5 mg MSU for the indicated times. Protein lysates were subjected to immunoblot analysis for phosphorylated IκB (P-IκB) or IκB. As a positive control for IκB phosphorylation, OBs were stimulated with TNF-α (50 ng/ml) for the indicated times.

MSU microcrystals ingested by macrophages have been shown to stimulate the production of IL-1β through the NLRP3 inflammasome [9]. Because NLRP3 is expressed by OBs [44], we examined next whether MSU in OBs is capable of activating the NLRP3 inflammasome. As a first step, we investigated whether IL-1β was produced by OBs in the presence of 0.5 mg MSU/10^6^ cells for 24 and 48 hours of culture. No extracellular IL-1β (as evaluated with EIA) or intracellular pro-IL-1β (as evaluated with immunoblot), even in the presence of 1 m*M* ATP, which activates NLRP-3 [77], was detected in MSU-stimulated OBs (data not shown). However, OBs exposed to MSU increased their expression of NLRP3 protein, which peaked at 12 hours of MSU stimulation and decreased after 24 hours, as evaluated with densitometry (Figure 6A, B).

Conversely, NF-κB is activated by solid particles ingested by OBs and by MSU in monocytic cells [20,28]. Its activation was assessed through the kinetic phosphorylation of its inhibitor IκB in OBs in the presence of MSU. No modification of IκB phosphorylation was detected in OBs activated by MSU, whereas TNF-α addition to OBs was typically associated with changes of IκB phosphorylation (Figure 6C). Overall, these results indicate that OBs respond to MSU (a) by a primary nonconventional phagocytosis followed by (b) a secondary autophagy, (c) by activating NLRP3 protein without concomitant IL-1β generation, and (d) by no signal through the NF-κB pathway.

### MSU-stimulated autophagy is regulated by NLRP3

**Figure 7:**
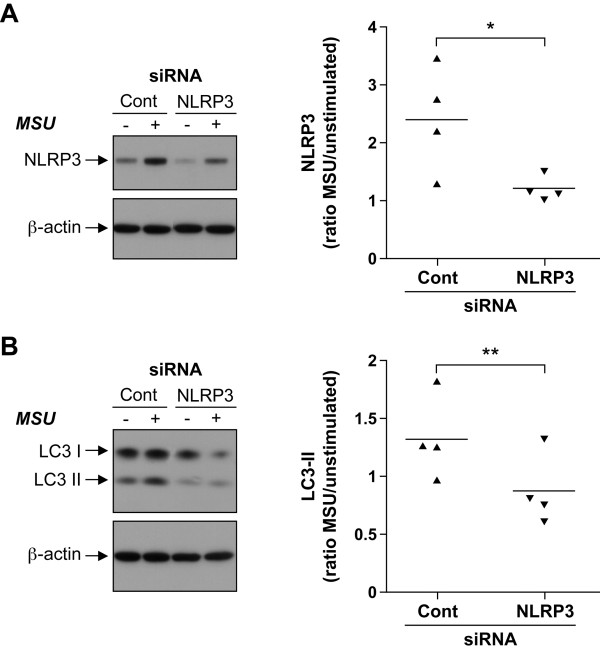
**NLRP3 upregulates MSU-induced autophagy.** OBs were transfected with AllStars Negative Control siRNA or NLRP3-specific siRNAs for 24 hours and then left unstimulated or stimulated with 0.5 mg MSU for 8 hours. **(A)** Immunoblot analysis using anti-NLRP3 and anti-β-actin antibodies. Densitometric analyses of NLRP3 expression. Data are expressed as arbitrary densitometric units normalized by β-actin levels; NLRP3 was presented as the ratio of NLRP3 levels of MSU-stimulated cells to NLRP3 levels of vehicle-stimulated cells. Statistical analysis was performed by using the paired two-tailed *t* test (*n* = 4 different donors). **(B)** Immunoblot analysis using anti-LC3 and anti-β-actin antibodies. Data are expressed as arbitrary densitometric units normalized by β-actin levels; LC3-II conversion induced by MSU was the ratio of LC3-II levels of MSU-stimulated cells to LC3-II levels of vehicle-stimulated cells. Results are expressed as mean ± SEM (*n* = 4 different donors). Statistical analysis was performed by using the paired two-tailed *t* test; *P < 0.05; **P < 0.001.

Under certain conditions like bacterial infection of macrophages, another inflammasome, the NLRC4/Ipaf inflammasome, has been reported to downregulate autophagy independent of IL-1β production [78]. In addition, members of the NLR protein family, like NOD1 and NOD2, are intracellular sensors that induce autophagy independent of NF-κB [79,80]. Could NLRP3 be implicated in the regulation of autophagy activated by MSU in OBs? To determine the role of NLRP3 in MSU-mediated autophagy, siRNAs were used to knockdown the expression of NLRP3 in OBs. Transfection of OBs with a combination of two NLRP3-specific siRNAs inhibited by 44% ± 9% the NLRP3 expression activated by MSU (Figure 7A). In addition, the LC3-II cleavage induced by MSU was decreased by 23% ± 1% in NLRP3 knockdown OBs (Figure 7B). These results indicate that NLRP3 activated by MSU in OBs is implicated in the upregulation of autophagy.

## Discussion

NLRP3 belongs to the family of cytosolic NLR proteins that help respond to a danger by recognition of bacterial particles, chemicals, and products from injured cells. Once activated, NLRP3 proteins associate with other cytosolic proteins to form an inflammasome presently known as a pivotal structure in the inflammatory process and in diseases in which IL-1β is greatly involved. NLRP3 activation is a hallmark of professional phagocytes involved in the immune responses. However, nonprofessional phagocytes also express NLRP3. Interestingly, two members of the NLR protein family, the intracellular sensors nucleotide-binding oligomerization domain-containing protein-1 and −2, are already coupled to autophagy [80].

Here, we identify a new role for another NLR protein, NLRP3, as a positive regulator of autophagy in response to the danger-signal MSU in human OBs. The functional relevance of this mechanism was shown by knockdown of NLRP3 and by blocking the process of MSU phagocytosis, which both led to the absence of cleavage of LC3-II. Thus, MSU provoked in OBs two different patterns of activation that appear closely related, an initial and necessary event of phagocytosis followed by a rapid induction of autophagy with the appearance of autophagosomes, conditions that together should lead to the complete removal of MSU. One of the major functions of autophagy through tightly controlled formation of autophagosomes is devoted to the removal of particles that escape degradation in conventional phagosomes [81,82]. However, the present results indicate that both primary processes of phagocytosis and autophagy in OBs are not followed by the degradation of internalized MSU microcrystals that remain intact inside persistent autophagosomes. In addition, survival of OBs is not affected by MSU, but their proliferation is reduced.

Our present results of the absence of MSU effect on OB mortality seems apparently in contradiction to a previous study that reported an inhibition of OB viability by MSU [83]. However, major experimental differences between this report and the present study can explain this discrepancy. The experiments presented here were performed with primary human OBs only, whereas Chhana’s studies [83] were carried out mostly with murine MC3T3-E1 cells, and the only viability data with human primary OBs of the published report used the MTT assay, which is, at best, an assay evaluating cell proliferation and that requires controlling several important parameters, to be an indirect test of cell viability [84]. Moreover, in the present study, we evaluate only PI incorporation by OBs, which represents a useful quantification of necrotic and late apoptotic cells [56]. Interestingly, although OB proliferation is reduced by MSU, their catabolic functions are activated because they are always alive after 7 days of culture. The absence of degradation of MSU by these nonprofessional phagocytes was corroborated with the findings of MSU directly encrusted in the irregular matrix of gouty lesions of bone [29].

Although visualization of MSU inside vacuoles was delayed for up to 24 hours, and NLRP3, which precedes the cleavage of LC3-I into LC3-II, appeared within 3 hours in MSU-stimulated OB, intracellular signaling indicated a rapid activation of both autophagy and phagocytosis. Moreover, the process of phagocytosis appeared an absolute necessity for subsequent autophagy of MSU, as shown by the absence of MSU autophagy secondarily to phagocytosis blockade. These sequences of phagocytosis followed by autophagy seem logical, because autophagy is aimed at destroying intracellular particles, whereas phagocytosis, also aimed at degrading foreign particles, is the process that will internalize extracellular particles. However, phagocytosis could have been sufficient to destroy MSU. Interestingly, MSU in the presence of OBs, nonprofessional phagocytes, can act as a danger signal and trigger the autophagy process through the rapid induction of NLRP3 to complete the degradation of MSU. It has been reported that autophagy participates in degrading extracellular microorganisms linking autophagy to phagocytosis [30,85]. It is also important to stress that NLRP3 activated by MSU in OBs does not engage the inflammasome signaling pathways, as it does in professional phagocytes [77], because expression of the adaptor ASC was not increased, and no activation of caspase-1 was detected in MSU-stimulated OBs (data not shown). Our results demonstrate that NLRP3 has an inflammasome-independent, cell-intrinsic effect in OBs ingesting MSU microcrystals.

MSU interaction with OBs also seems particularly original at the level of kinetics and regulation of phagocytosis. First, engulfment of MSU by OBs is related to a process of phagocytosis, because cytochalasin D blocked entirely MSU internalization, whereas colchicine, an inhibitor of microtubule polymerization, had no effect. OBs can ingest various foreign particles like MSU, titanium (Ti), latex beads, or microorganisms like *Escherichia coli* or *Candida albicans*[19,86]. However, Ti, for instance, activates NF-κB in OBs [20], whereas MSU did not. Moreover, this difference of signals involved in MSU phagocytosis is also demonstrated at the level of Src kinases required for phagocytosis by professional phagocytes [87], whereas they are not required by OBs. In addition, ERK1/2 and p38 MAPK, which positively regulate conventional phagocytosis, have opposite effects in MSU-activated OBs as human phosphokinase array revealed a phosphorylation of p38 MAPK, but SB203580, an inhibitor of p38, did not reduce but facilitated phagocytosis. Our results suggest that phagocytic stimulation by MSU required ERK activation but not p38, which seems to act as a repressor of MSU phagocytosis by OBs. Such antagonistic roles of ERK1/2 and p38 MAPK are reminiscent of another condition in which ERK1/2 and p38 MAPK differentially regulate heme biosynthesis [88].

The primary function of both phagocytosis and autophagy is to maintain cellular homeostasis by degrading foreign particles that can represent successively an extracellular danger and, after their ingestion, another intracellular danger, if phagocytosis failed in its function of destruction. Interestingly, even if extracellular MSU crystals present a major proinflammatory potential, they have been recognized as an endogenous danger signal useful to immunity [6]. From the presence of such MSU crystals in cells and tissues emerges the concept of their degradation. It is well known that an attack of gout can spontaneously improve and MSU crystals remain present in joints and tissues. MSU deposits can be shown in various tissues from the joint to cartilage, bone, vasculature, skin, and kidney. It seems that once crystallized in humans, MSU cannot be easily and spontaneously degraded. Our results seem to confirm that notion, at least in bone tissues.

The difference between professional and nonprofessional phagocytes relies on, at least in part, their rapidity and efficiency of phagocytosis [89]. Although neutrophils rapidly ingest MSU *in vitro*, only a few neutrophils are shown with intracellular microcrystals, and these neutrophils rapidly die with release of cellular content [90,91]. Macrophages poorly ingest MSU microcrystals that have, however, profound stimulatory effects on these phagocytes [92,93]. In contrast, most of the OBs that slowly vacuolize microcrystals, ingested MSU but did not die from this process. Moreover, OBs in contact with MSU crystals rapidly stimulate signaling of phagocytosis and NLRP3 for their subsequent autophagy, both mechanisms of particle destruction that fail in MSU degradation. OBs with MSU crystals inside did not die, but showed profound changes of their functions, becoming bone cells that have reduced capacity of mineralization, that degrade the calcified matrix, but that have no change of RANKL and OPG mRNAs (RT-PCR data not shown). Also, the upregulation of autophagy by NLRP3 in these conditions did not generate IL-1β, although mammalian cells can produce IL-1β via an autophagy-based secretory pathway [94]. However, the absence of IL-1β production by OBs could also be related to their incapacity to translate mRNA, as reported for OB phagocytosis of *Staphylococcus aureus* and *Salmonella*[95]. Thus, the process of autophagy activated by MSU in OBs could partly detoxify these cells by retaining MSU microcrystals in permanent autophagosomes.

## Conclusion

MSU crystals in the presence of the nonprofessional phagocytes OB selectively activate the MAPK pathways, without any effect on NF-κB and Src kinases, leading successively to the two primary processes of degradation of foreign particles that penetrate inside the cell, phagocytosis and autophagy. However, despite a rapid upregulation of autophagy through NLRP3, MSU microcrystals remain intact inside OBs that do not affect their survival but reduce their proliferation. The present osteoblastic consequences of MSU ingestion are profound modifications of their functional phenotype that, in the context of bone tissues in gout, validate the pathologic findings of MSU microcrystals remaining encrusted in bone. Hence, NLRP3 could upregulate autophagy in other pathologic conditions and could have an important function in diseases.

## Abbreviations

LC3: Microtubule-associated protein light chain 3; MMP: matrix metalloproteinase; MSU: monosodium urate; NLRP: nucleotide-binding domain and leucine-rich repeat region-containing family of receptor protein; OB: osteoblast; PKC: protein kinase C; Syk: spleen tyrosine kinase; TOR: target of rapamycin.

## Competing interests

All authors state that they have no competing interests.

## Authors’ contributions

Study design was by IA and PEP, and the study was conducted by IA. Data analysis was done by IA, FM, and PEP. Reagents/materials/analysis and tools were contributed by PEP and FM. IA and PEP drafted the manuscript. Revising manuscript content was done by IA, FM, and PEP. PEP takes responsibility for the integrity of the data analysis. All authors read and approved the final manuscript.
